# Classification and prevalence of spin in abstracts of non-randomized studies evaluating an intervention

**DOI:** 10.1186/s12874-015-0079-x

**Published:** 2015-10-13

**Authors:** Clément Lazarus, Romana Haneef, Philippe Ravaud, Isabelle Boutron

**Affiliations:** METHODS Team, Centre of Research in Epidemiology and Statistics Sorbonne Paris Cité (CRESS), UMR 1153, INSERM, Paris, France; Paris Descartes University, Sorbonne Paris Cité, Faculté de Médecine de Paris, Paris, France; Centre d’Épidémiologie Clinique, Hôpital Hôtel Dieu, Assistance Publique des Hôpitaux de Paris, 1, place du Parvis Notre-Dame, 75181 Paris, France; French Cochrane Center, Paris, France; Department of Epidemiology, Columbia University Mailman School of Public Health, New York, NY USA

## Abstract

**Background:**

Spin represents specific reporting strategies, either intentional or unintentional, to convince the reader that the beneficial effect of the experimental intervention in terms of efficacy and safety is greater than that shown by the results. The objectives of this study were to 1) develop a classification of spin specific to non-randomized studies assessing an intervention and 2) estimate the prevalence of spin in abstracts of reports of such studies.

**Methods:**

In a first step, we developed a specific classification of spin for non-randomized studies by a literature review and pilot study. In a second step, 2 researchers trained in the field of methodology evaluated the prevalence of spin in the abstract of all non-randomized studies assessing an intervention published in the BioMed Central Medical Series journals between January 1, 2011 and December 31, 2013. All disagreements were resolved by consensus. We also determined whether the level of spin in abstract conclusions was high (spin reported without uncertainty or recommendations for further trials), moderate (spin reported with some uncertainty or recommendations for further trials) or low (spin reported with uncertainty and recommendations for further trials).

**Results:**

Among the 128 assessed articles assessed, 107 (84 %) had at least one example of spin in their abstract. The most prevalent strategy of spin was the use of causal language, identified in 68 (53 %) abstracts. Other frequent strategies were linguistic spin, inadequate implications for clinical practice, and lack of focus on harm, identified in 33 (26 %), 25 (20 %), and 34 (27 %) abstracts respectively. Abstract conclusions of 61 (48 %) articles featured a high level of spin.

**Conclusion:**

Abstract of reports of non-randomized studies assessing an intervention frequently includes spin. Efforts to reduce the prevalence of spin in abstract for such studies are needed.

**Electronic supplementary material:**

The online version of this article (doi:10.1186/s12874-015-0079-x) contains supplementary material, which is available to authorized users.

## Introduction

Spin, or distortion of study findings, can be used by authors to enhance their study findings more than the results justify [1, 2]. Recent studies demonstrated a high prevalence of spin in study reports. Spin was found in more than half of the abstract conclusions of randomized controlled trials with statistically non-significant results for the primary outcome [3]; moreover, one third of reports of diagnostic accuracy studies contained a form of over-interpretation [4]. The spin used consisted mainly of a focus on statistically significant results (within-group comparison, secondary outcomes, subgroup analyses, modified population of analyses); or interpreting statistically non significant results for the primary outcomes as showing treatment equivalence or comparable effectiveness. A recent study in the field of cancer found that the prevalence of spin has increased over time [5]. In the same field, a randomized controlled trial demonstrated that spin in abstracts could modify a reader’s interpretation of study results [6].

Non-randomized studies are commonly used in medical research to evaluate interventions. They are particularly useful to draw conclusions about the safety or efficacy of interventions in real-world settings, to assess rare or long-term adverse events or when randomization is not possible (e.g., surgical procedures). However, these designs have important limitations. Non randomized designs are also susceptible to many type of spin which could be same or different from those previously described [7]. Particularly, contrary to randomized clinical trials, they may not allow for establishing causal inferences but rather, only an association [8–10].

Our study aimed to 1) develop a classification of spin for non-randomized studies assessing therapeutic interventions and 2) estimate the prevalence of spin in the abstracts of non-randomized studies evaluating a therapeutic intervention published in BioMed Central Medical Series journals.

## Methods

### Development of a classification of spin

Spin was defined as the use of specific reporting strategies, either intentional or unintentional, to convince the reader that the beneficial effect of the experimental treatment in terms of efficacy and safety is higher than is actually shown by the data.

To develop the classification of spin, we performed a literature review of studies of spin for other study designs [3–5, 9, 11] as well as studies of distorted presentation and interpretation of findings from non-randomized studies [12–19]. From these data, we developed a preliminary classification, discussed among the authors and tested by two 2 researchers (CL and RH) with a sample of 15 articles. The classification was discussed until consensus was achieved among the authors.

### Prevalence of spin in abstracts

We selected a sample of reports of non-randomized studies evaluating an intervention published in 25 journals of the BioMed Central Medical Series that regularly publish clinical studies. We selected these journals because they are open-access and publish reports of non-randomized studies evaluating therapeutic interventions from a large range of medical specialties.

### Search strategy

We searched MEDLINE via PubMed (search date January 21, 2014) for all articles published in the 25 BioMed Central Medical Series journals between January 1, 2011 and December 31, 2013. The list of selected journals and complete search strategy are respectively in Additional files 1 and 2.

### Study identification

**Fig. 1:**
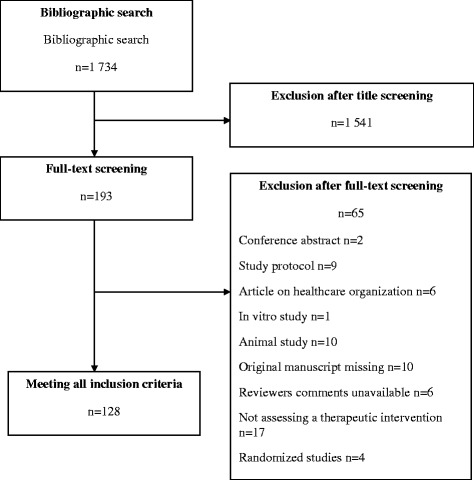
Flowchart for the selection and inclusion of articles from the BioMed Central Medical Series Journals assessing therapeutic interventions through non-randomized designs and reasons for exclusion

One researcher (CL) screened all titles, abstracts and, if necessary, the full-text articles of the citations retrieved and selected all reports of non-randomized studies assessing a therapeutic intervention defined as a pharmacological or non-pharmacological treatment (e.g., pharmaceutical drugs, surgery, therapeutic education, rehabilitation, paramedical care etc.) proposed to patients to improve their health. We excluded medico-economic assessments of therapeutic interventions and protocols of observational studies (Fig. 1). As a quality control, a second trained researcher (RH) assessed a random selection of 10 % of articles retrieved by the bibliographic search to ensure that some articles were not missed. This second researcher did not retrieve any missed articles in this subset. For every included article, we retrieved the full-text article and the abstract.

### Data extraction

For each selected article, 2 researchers (CL and RH) trained in the field of methodology independently read the abstract and full-text article and collected data on the general characteristics of the study–study design, sample size, type of therapeutic intervention, comparator, funding sources and whether registration was recorded–using a standardized data-extraction form. They systematically searched for spin in the abstracts using the classification system developed previously.

### Level of spin in abstract conclusions

The 2 researchers evaluated the level of spin in the abstract conclusions. A low level of spin was defined as spin reported with uncertainty in the framing and recommendations for further trials, a moderate level as spin reported with some uncertainty in the framing or recommendations for further trials, and a high level as spin reported without any uncertainty or recommendations for further trials.

Any discrepancies were solved by consensus and, if needed, by consultation with a third researcher (IB).

### Statistical analysis

Data are reported with median (Q1–Q3) for continuous variables and number (%) for categorical variables. Statistical analyses involved use of R 2.15.0 (http://www.R-project.org, the R Foundation for Statistical Computing, Vienna, Austria) and any appropriate packages.

## Results

### Selected articles

The search strategies retrieved 1,734 citations; 128 were selected and assessed. The description of included articles is in Table 1. The study designs of the selected articles were prospective cohort studies (n = 42, 33 %), historical cohort studies (n = 39, 30 %) and before–after studies (n = 40, 31 %). The interventions evaluated were drugs (n = 51, 40 %), non-pharmacologic interventions (e.g., surgery, device or equipment, behavioral intervention or participative; n = 48, 38 %), and therapeutic strategy (n = 29, 23 %). The median [Q1–Q3] sample size was 130 [51–458]. The funding source was mainly non-profit (n = 54, 42 %), but for 41 articles (32 %) the funding source was not reported or was unclear.

**Table 1:** Characteristics of included articles

Characteristics	*n* = 128 (%)
Study design	
Prospective	42 (33 %)
Before–after	40 (31 %)
Historical cohort	39 (30 %)
Cross-sectional	6 (5 %)
Other	1 (1 %)
Therapeutic intervention	
Drug	51 (40 %)
Surgery	14 (11 %)
Device or equipment	14 (11 %)
Behavioral intervention or participative	20 (16 %)
Therapeutic strategy	29 (23 %)
Comparator	
Placebo or attention control intervention	4 (3 %)
Active treatment	55 (43 %)
Usual care or no treatment	25 (20 %)
No comparator or Unclear	44 (34 %)
Sample size, median [Q1–Q3], (range)	129.5 [50.75–457.5], (5–238600)
Funding source	
Profit	28 (22 %)
Not for profit	54 (42 %)
Both	5 (4 %)
Not reported or unclear	41 (32 %)
Registration	
Yes	39 (30 %)
No or not reported	89 (70 %)

### Classification of spin

The classification of spin we developed was divided into 3 categories: misleading reporting, misleading interpretation and inadequate extrapolation of the results. Each of these categories included several spin strategies, which are detailed below. Table 2 provides a clear definition of each spin category with an example of spin:

**Table 2:** Spin classification for non-randomized studies assessing therapeutic interventions. Examples provided are from manuscripts and published abstract and full-texts from our sample

Category of spin	Strategy used	Definition	Example
Misleading reporting	Not reporting adverse events or lack of focus on harm	Results are reported without warnings on important or relevant safety issue.	“This trial showed that overnight switch from oral ropinirole to transdermal rotigotine, with a dose conversion ratio of 1.5:1, was well tolerated in Korean patients with no apparent loss of efficacy.” 45/116 experienced adverse events and 13 stopped treatments because of adverse events.
	Selective reporting	Only a subset of the original outcomes or analysis planned in a study is fully reported.	“The results suggest that [Internet-based cognitive therapy] ICBT with therapist support has the potential to reduce [obsessive-compulsive disorders] OCD symptoms, depressive symptoms and general functioning”. No report of the lack of improvement in quality of life.
	Misleading description of study design	Study design is presented as more robust than it is actually.	“Based on this prospective case control study tranexamic acid seems not to have a benefit in posterior lumbar spine surgery.” It was a retrospective study involving 97 patients and nothing was prospective in this study.
	Use of linguistic spin	Any word or expression emphasizing the beneficial effect of the therapeutic intervention	“Both operative methods could be performed safely in the early stages after the introduction of surgery and could consistently obtain excellent surgical performance.”
	No consideration of the limitations	Important limitations are not taken into account in the interpretation of the results.	“The TABADO program, targeting teenagers in vocational schools, was effective in producing a higher 12-month abstinence rate among all smokers in the intervention group.” No adjustment was made on major confounding variables.
	Selective citation of other studies	Only previous studies concordant with the current study findings are acknowledged or other important studies in the field are not reported.	“It would be interesting to know its efficacy and safety in correcting high myopic astigmatism and how it changes the shape of the cornea.” Several publications already exist in this field.
Inadequate interpretation	Claim an effect for non-statistically significant results	Therapeutic intervention is presented as effective despite a non-statistically significant result.	“The use of [Automated CardioPulmonary Resuscitation] A-CPR resulted in a higher rate of survival to hospital compared with [Conventional CardioPulmonary Resuscitation] CPR” in a restrospective study involving 66 patients where the propensity score adjusted Odds Ratio was 1.69 [0.79; 3.63].
	Claim an equivalence for non-statistically significant results despite a wide confidence interval	Therapeutic intervention and comparator are presented as equivalent when a comparison test is not statistically significant with a large confidence interval.	“The authors concluded that: 1) mortality during follow-up was statistically similar for both groups; (…)” In this retrospective cohort study involving 352 patients, the survival rate at 20 year was 69.3 % with mechanical mitral valve substitutes and 56.6 % with biological substitutes. The hazard ratio was not statistically significant but had a wide 95 % confidence interval (HR = 1.21 [0.79; 1.86], *p* = 0.386).
	Ruling out safety for non-statistically significant results	Therapeutic intervention is presented as safe based on a non-statistically significant comparison test, despite a large confidence interval.	“Long-term treatment with esomeprazole (20 mg once daily) is well tolerated and efficacious” despite 16.29 % of patients (*n* = 22) experiencing adverse events judged to be possibly related to treatment with esomeprazole (mostly mild and transient).
	Causal language or causal claim	Results are presented with a sentence implying a cause-and-effect link between the intervention and the outcome	“Treatment with oral valacyclovir as the sole antiviral therapy resulted in complete resolution of retinitis.” This was a before–after study involving 10 patients.
	Claim of any significant difference despite lack of statistical test	Therapeutic intervention and comparator are compared despite no proper statistical test reported.	“pH and HCO^3−^ significantly increased after hemodialysis sessions with both aspirin only and aspirin + dipyramidol, the increase of pH and HCO^3−^with aspirin only was significantly larger than aspirin + dipyramidol.” No statistical test was reported.
	Focus on statistical significance instead of clinical relevance	Results are presented by their statistical significance without considering the clinical relevance of the effect size.	“While the [Clinical Global Impression-Schizophrenia] CGI-SCH overall score improved in both groups after switching, there was a significantly greater change in those who switched from olanzapine (difference of 0.29 points, *p* = 0.013)”. The CGI-SCH scale range from 0 to 7.
Inadequate extrapolation	Inadequate extrapolation to larger population, intervention or outcome	Results are generalized to another population, intervention or outcome than those of the study (such as surrogate outcomes)	“This intervention approach has the potential to impact on the progression of colorectal cancers and other cancers or chronic diseases.” The intervention focused on colorectal cancers only.
	Inadequate implication for clinical practice	Authors recommend the use of the therapeutic intervention for clinical practice.	“One should not hesitate to perform an osteotomy in difficult cases.” in a prospective cohort study involving 109 patients.

- Misleading reporting of the results was defined as incomplete reporting of the study results that could be misleading for the reader. This type of spin included 1) not reporting adverse events or lack of focus on harms (e.g., no warning on important safety issues), 2) selective reporting of outcomes favoring the beneficial effect of the experimental treatment (e.g., statistically significant results for efficacy outcomes or statistically non-significant results for harm outcomes), 3) misleading reporting of study design, 4) use of linguistic spin or “hype” (i.e., rhetorical manipulations to convince the readers of the beneficial effect of the treatment such as “excellent” results, “encouraging” outcome*s*, “a trend toward significance”), 5) no consideration of limitations, and 6) selective citation of other studies.
- Inadequate interpretation of the results was defined as misleading interpretation of the study results overestimating the beneficial effect of the intervention. This type of spin included 1) claiming a beneficial effect of the intervention despite statistically non-significant results, 2) claiming an equivalent effect of the interventions for statistically non-significant results despite wide confidence interval, 3) claiming that the treatment is safe for statistically non-significant safety outcomes despite lack of power, 4) concluding a beneficial effect despite no comparison test performed, 5) interpretation of the results according to statistical significance (p-value) instead of clinical relevance, or 6) claiming a causal effect between the intervention being assessed and the outcome of interest despite a non-randomized design. Use of causal language was defined as any statement addressing the causal relationship of the intervention and outcomes with 1) modal auxiliary verbs, with the intervention as the subject and the outcome as a direct object (e.g., “Surgical experience could shorten the duration of TVT [tension-free vaginal tape] surgery.” [20]); 2) use of terms belonging to the semantic field of causal relationship (e.g., “effective”, “improve”, “enhance”); or 3) use of a tone inferring a strong result (e.g., “The results demonstrate” or “This study shows that”). We did not consider that causal language was used when authors stated only a co-occurrence between the intervention and the outcome (e.g., “subjects with symptomatic bipolar disorders who relapse frequently showed improvements in each of these areas after treatment with RLAI [risperidone long-acting injection]” [21]). We did not consider causal language as spin in studies using a propensity score or instrumental variables [22].
- Inadequate extrapolation of the results was defined as an inappropriate generalization of the study result by inadequate 1) extrapolation from the population, interventions or outcome actually assessed in the study to a larger population, different interventions or outcomes, or 2) inadequate implications for clinical practice.

### Prevalence of spin in abstracts

In total, 107 (84 %) reports had at least one type of spin in their abstracts (Table 3). The median number of type of spin per abstract identified was 2 (Q1–Q3 1–3, range 0–6). The most prevalent spin strategy related to the use of causal language identified in 68 (53 %) abstracts. For example, in a before–after study including 7 patients, the authors stated “Erythropoietin … *increases* the oxygen partial pressure in the brain tissue … in poor grade SAH [subarachnoid aneurismal hemorrhage] … patients with severe cerebral vasospasm” [23] and in another prospective study including 22 patients, they stated “[Bi-level positive airway pressure-spontaneous/timed] BiPAP S/T with AVAP [average volume assured pressure support] … *facilitates rapid recovery* of consciousness when compared to traditional BiPAP S/T in patients with chronic obstructive pulmonary disease and hypercapnic encephalopathy” [24].

**Table 3:** Spin in the abstracts of published articles

Spin categories	Presence of at least one example of spin in the abstract
	*n* = 128
At least one spin in the abstract	107 (84.0)
Misleading reporting	
Not reporting adverse events or lack of focus on harm	34 (26.6)
Selective reporting	15 (11.8)
Misleading description of study designs	1 (0.8)
Use of linguistic spin or “hype”	33 (25.8)
Inadequate interpretation	
Claim an effect for non-statistically significant results	7 (5.5)
Claim equivalence for non-statistically significant results	13 (10.2)
Ruling out safety when results are not statistically significant	15 (11.8)
Causal language or causal claim	68 (53.1)
Claim any difference despite no comparison test performed	1 (0.8)
Focus on statistical significance instead of clinical relevance	1 (0.8)
Inadequate extrapolation	
Inadequate extrapolation to larger population, intervention or outcome	11 (8.6)
Inadequate implications for clinical practice	25 (19.5)
Other	5 (3.9)

Data are no. (%)

Other frequent strategies of spin were linguistic spin, inadequate implications for clinical practice and lack of focus on harms, in 33 (26 %), 25 (20 %), and 34 (27 %) abstracts, respectively. For example, we considered linguistic spin frequent when authors indicated that the results were close to significance, despite a p-value > 0.05. For example, in a prospective study including 662 patients, the authors stated “a tendency towards lower all-cause mortality at 3 months with use of Aspirin + dipyramidol” (p = 0.12) [25] and others used superlatives or “hype” to highlight a beneficial effect of the intervention assessed (e.g., “high potential,” “considerably helps,” “excellent results”). Inadequate implications for clinical practice occurred mainly when authors extrapolated some recommendations for clinical practice from their results (e.g., in a retrospective study of 42 patients: “it is a suitable therapeutic option not only for initial drainage but also for salvage therapy” [26]).

We identified selective reporting in more than 12 % of abstracts, (e.g., in a before–after study of 23 patients: “[Internet-based cognitive behavior therapy] ICBT… with therapist support reduces [obsessive-compulsive disorder] OCD symptoms, depressive symptoms and improves general functioning” [27], with no report of the lack of improvement in quality of life found in this study). Also, in 13 % of abstracts, authors concluded on the safety of the intervention solely by a statistically non-significant difference in safety outcome despite lack of power. For example, in a historical cohort of 54 patients, the authors reported “Intravenous sodium valproate is as effective as intravenous phenytoin as the first-line treatment in status epilepticus … with no significant cardiovascular compromise” [28], despite more than twice as many deaths in the intravenous penytoin group (30 % vs 11 %) although not statistically significant.

### Level of spin in abstract conclusions

We classified 61 articles (48 %) as containing a high level of spin in abstract conclusions, 24 (19 %) a moderate level of spin and 17 (13 %) a low level of spin. Only 26 articles (20 %) did not have spin in their abstract conclusions.

## Discussion

To our knowledge, this is the first study to develop and use a classification of spin for non-randomized studies. Of the 128 reports we evaluated, 84 % of abstracts contained at least one type of spin and 48 % featured a high level of spin. Use of causal language was the most frequent spin strategy. Yet, the use of causal claims is misleading in the interpretation of non-randomized studies because these designs are unable to control for every confounding factor.

Our findings are consistent with results of other studies. Many of the spin strategies we observed are shared with those identified in randomized controlled trials. Spin strategies are varied and frequent and might lead to misleading interpretation of the study results. This situation is problematic because it has been demonstrated [6] that spin in the abstract conclusions of randomized controlled trial reports could bias the interpretation of the results by clinicians [29].

A particular feature of our work was the important prevalence of causal claims in abstracts of non-randomized studies. Causal language is a specific spin strategy for non-randomized studies. Such designs, as opposed to randomized studies, do not allow for concluding a cause-and-effect link between the assessed intervention and the observed outcome but rather, provide information only about association [8, 30, 31]. Some studies have explored the causal language in such designs. Cofield *et al*. [30] observed a 31 % rate of causal language in a series of 525 peer-reviewed papers on obesity and nutrition. Brown *et al.* [32] evidenced a 26 % to 50 % rate of studies ascribing greater inferential strength than the study design warranted. Causal claims in epidemiology are a cornerstone of the interpretation of results [33], especially for complex interventions for which randomization is difficult or impossible [11]. In 2012, journal editors of the HEART Group published an editorial review about “the importance of matching language to type of evidence” [34] and concluded with a plea to investigators and editors to “carefully select language used during reporting to match the type of study conducted.” The rating of causal inference by researchers was assessed in a study of 38 randomized clinical trials and 35 non-randomized clinical trials [35]. The results highlighted that authors “might have overstated the strength of causal inference in the abstracts of non-randomized clinical trials, but appeared to report causality appropriately in the main text.”

Our study has several limitations. First, our sample is not representative of all non-randomized studies assessing therapeutic interventions indexed in PubMed. We choose the BioMed Central series of medical journals because it is an open-access and open peer-review collection involving a wide variety of medical specialties, therapeutic interventions and study designs. These journals are also strongly involved in the requirement of reporting guidelines (STROBE, CONSORT) and transparency policies (ICMJE) from authors. Consequently, we cannot extrapolate our results to other journals. Second, the assessment of spin strategies is subjective because the interpretation of the results highly depends on the context. To address this issue, 2 trained researchers independently collected the data using a standardized form, with discrepancies resolved by consensus and the involvement of a third researcher if necessary. Third, our study could not determine whether the spin strategies were conscious attempts to show the treatment as more beneficial than it actually was. We did not assess the impact of spin in abstracts of non-randomized studies on the interpretation of such studies, which can differ depending on the category of spin considered. It is important to recognize that an abstract may contain some spin item but actually be balanced overall. We attempted to take into account this issue and assessed the level of spin in the abstracts conclusions provided some elements about the importance of uncertainty and call for further research for the global tone of a conclusion.

Further studies should be conducted to determine the impact of spin on the readers’ interpretation of the study results. It is possible that the presence of spin item could be counterbalanced by other element in the reporting of abstracts.

## Conclusion

We found a high prevalence of spin in abstracts of reports of non-randomized studies. Misleading interpretation of results of such studies could lead to inadequate clinical practices and erroneous beliefs in the effects of therapeutic interventions. The classification we developed should facilitate efforts to reduce the prevalence of spin.

## Additional files

Additional file 1:
**Selected journal from the BioMed Central medical journals series.** (PDF 7 kb) Additional file 2:
**Complete bibliographic request.** (PDF 10 kb)

## Abbreviations

(Q1–Q3): Interquartile Range
A-CPR: Automated CardioPulmonary Resuscitation
AVAPS: Average Volume Assured Pressure Support
BiPAP S/T: Bi-level Positive Airway Pressure–Spontaneous / Timed
CGI-SCH: Clinical Global Impression-Schizophrenia
CPR: CardioPulmonary Resuscitation
HR: Hazard Ratio
ICBT: Internet-based Cognitive Behavior Therapy
OCD: Obsessive–Compulsive Disorder
RLAI: Risperidone Long-Acting Injection
SAH: Sub-Arachnoïdal Hemorrhage
TVT: Tension-free Vaginal Tape
