# Supplementary material for: Classification and prevalence of spin in abstracts of non-randomized studies evaluating an intervention
Source: BMC Med Res Methodol. 2015 Oct 13;15:85. doi: 10.1186/s12874-015-0079-x (PMC4604617; doi:10.1186/s12874-015-0079-x)
Supplement: Additional file 1: — Selected journal from the BioMed Central medical journals series. (PDF 7 kb) [file 12874_2015_79_MOESM1_ESM.pdf]

Selected journal from the BioMed Central medical journals series

- ✓ BMC Cancer
- ✓ BMC Cardiovascular Disorders
- ✓ BMC Clinical Pharmacology
- ✓ BMC Complementary and Alternative Medicines
- ✓ BMC Dermatology
- ✓ BMC Emergency Medicine
- ✓ BMC Family Practices
- ✓ BMC Gastroenterology
- ✓ BMC Geriatrics
- ✓ BMC Health services research
- ✓ BMC Infectious diseases
- ✓ BMC Medicine
- ✓ BMC Musculoskeletal disorders
- ✓ BMC Nephrology
- ✓ BMC Neurology
- ✓ BMC Ophtalmology
- ✓ BMC Oral Health
- ✓ BMC Pediatrics
- ✓ BMC Pregnancy and childbirth
- ✓ BMC Psychiatry
- ✓ BMC Public health
- ✓ BMC Pulmonary medicine
- ✓ BMC Surgery
- ✓ BMC Urology
- ✓ BMC Women’s Health
