# Supplementary material for: Classification and prevalence of spin in abstracts of non-randomized studies evaluating an intervention
Source: BMC Med Res Methodol. 2015 Oct 13;15:85. doi: 10.1186/s12874-015-0079-x (PMC4604617; doi:10.1186/s12874-015-0079-x)
Supplement: Additional file 2: — Complete bibliographic request. (PDF 10 kb) [file 12874_2015_79_MOESM2_ESM.pdf]

Appendix 2 – Complete bibliographic request

((((("clinical trial"[Publication Type] OR "comparative study"[Publication Type] OR "controlled clinical trial"[Publication Type] OR)) NOT "randomized controlled trial"[Publication Type]) AND (("bmc bioinformatics"[Journal] OR "bmc biol"[Journal] OR "bmc biology"[Journal] OR "bmc biophys"[Journal] OR "bmc biophysics"[Journal] OR "bmc biotechnol"[Journal] OR "bmc biotechnology"[Journal] OR "bmc blood disord"[Journal] OR "bmc blood disorders"[Journal] OR "bmc cancer"[Journal] OR "bmc cardiovasc disord"[Journal] OR "bmc cardiovascular disorders"[Journal] OR "bmc cell biol"[Journal] OR "bmc cell biology"[Journal] OR "bmc chem biol"[Journal] OR "bmc chemical biology"[Journal] OR "bmc clin pathol"[Journal] OR "bmc clin pharmacol"[Journal] OR "bmc clinical pathology"[Journal] OR "bmc clinical pharmacology"[Journal] OR "bmc complement altern med"[Journal] OR "bmc complementary and alternative medicine"[Journal] OR "bmc dermatol"[Journal] OR "bmc dermatology"[Journal] OR "bmc dev biol"[Journal] OR "bmc developmental biology"[Journal] OR "bmc ear nose throat disord"[Journal] OR "bmc ear, nose, and throat disorders"[Journal] OR "bmc ecol"[Journal] OR "bmc ecology"[Journal] OR "bmc emerg med"[Journal] OR "bmc emergency medicine"[Journal] OR "bmc endocr disord"[Journal] OR "bmc endocrine disorders"[Journal] OR "bmc evol biol"[Journal] OR "bmc evolutionary biology"[Journal] OR "bmc fam pract"[Journal] OR "bmc family practice"[Journal] OR "bmc gastroenterol"[Journal] OR "bmc gastroenterology"[Journal] OR "bmc genet"[Journal] OR "bmc genetics"[Journal] OR "bmc genomics"[Journal] OR "bmc geriatr"[Journal] OR "bmc geriatrics"[Journal] OR "bmc health serv res"[Journal] OR "bmc health services research"[Journal] OR "bmc hematol"[Journal] OR "bmc hematology"[Journal] OR "bmc immunol"[Journal] OR "bmc immunology"[Journal] OR "bmc infect dis"[Journal] OR "bmc infectious diseases"[Journal] OR "bmc int health hum rights"[Journal] OR "bmc international health and human rights"[Journal] OR "bmc med"[Journal] OR "bmc med educ"[Journal] OR "bmc med ethics"[Journal] OR "bmc med genet"[Journal] OR "bmc med genomics"[Journal] OR "bmc med imaging"[Journal] OR "bmc med inform decis mak"[Journal] OR "bmc med phys"[Journal] OR "bmc med res methodol"[Journal] OR "bmc medical education"[Journal] OR "bmc medical ethics"[Journal] OR "bmc medical genetics"[Journal] OR "bmc medical genomics"[Journal] OR "bmc medical imaging"[Journal] OR "bmc medical informatics and decision making"[Journal] OR

"bmc medical physics"[Journal] OR "bmc medical research methodology"[Journal] OR "bmc medicine"[Journal] OR "bmc microbiol"[Journal] OR "bmc microbiology"[Journal] OR "bmc mol biol"[Journal] OR "bmc molecular biology"[Journal] OR "bmc musculoskelet disord"[Journal] OR "bmc musculoskeletal disorders"[Journal] OR "bmc nephrol"[Journal] OR "bmc nephrology"[Journal] OR "bmc neurol"[Journal] OR "bmc neurology"[Journal] OR "bmc neurosci"[Journal] OR "bmc neuroscience"[Journal] OR "bmc nucl med"[Journal] OR "bmc nuclear medicine"[Journal] OR "bmc nurs"[Journal] OR "bmc nursing"[Journal] OR "bmc ophthalmol"[Journal] OR "bmc ophthalmology"[Journal] OR "bmc oral health"[Journal] OR "bmc palliat care"[Journal] OR "bmc palliative care"[Journal] OR "bmc pediatr"[Journal] OR "bmc pediatrics"[Journal] OR "bmc pharmacol"[Journal] OR "bmc pharmacol toxicol"[Journal] OR "bmc pharmacology"[Journal] OR "bmc pharmacology toxicology"[Journal] OR "bmc physiol"[Journal] OR "bmc physiology"[Journal] OR "bmc plant biol"[Journal] OR "bmc plant biology"[Journal] OR "bmc pregnancy and childbirth"[Journal] OR "bmc pregnancy childbirth"[Journal] OR "bmc proc"[Journal] OR "bmc proceedings"[Journal] OR "bmc psychiatry"[Journal] OR "bmc public health"[Journal] OR "bmc pulm med"[Journal] OR "bmc pulmonary medicine"[Journal] OR "bmc res notes"[Journal] OR "bmc research notes"[Journal] OR "bmc sports sci med rehabil"[Journal] OR "bmc sports science, medicine and rehabilitation"[Journal] OR "bmc struct biol"[Journal] OR "bmc structural biology"[Journal] OR "bmc surg"[Journal] OR "bmc surgery"[Journal] OR "bmc syst biol"[Journal] OR "bmc systems biology"[Journal] OR "bmc urol"[Journal] OR "bmc urology"[Journal] OR "bmc vet res"[Journal] OR "bmc veterinary research"[Journal] OR "bmc women s health"[Journal] OR "bmc women's health"[Journal] OR "bmc womens health"[Journal]))) AND ("2011/01/01"[Date - Publication] : "2013/12/31"[Date - Publication]))
